# Comparative Phytochemical Characterization, Genetic Profile, and Antiproliferative Activity of Polyphenol-Rich Extracts from Pigmented Tubers of Different *Solanum tuberosum* Varieties

**DOI:** 10.3390/molecules25010233

**Published:** 2020-01-06

**Authors:** Luigi De Masi, Paola Bontempo, Daniela Rigano, Paola Stiuso, Vincenzo Carafa, Angela Nebbioso, Sonia Piacente, Paola Montoro, Riccardo Aversano, Vincenzo D’Amelia, Domenico Carputo, Lucia Altucci

**Affiliations:** 1 National Research Council (CNR), Institute of Biosciences and Bioresources (IBBR), Via Università 133, 80055 Portici (Naples), Italy; luigi.demasi@ibbr.cnr.it; 2 Department of Precision Medicine, University of Campania “Luigi Vanvitelli”, Via L. De Crecchio 7, 80138 Naples, Italy; paola.bontempo@unicampania.it (P.B.); paola.stiuso@unicampania.it (P.S.); vincenzo.carafa@unicampania.it (V.C.); angela.nebbioso@unicampania.it (A.N.); 3 Department of Pharmacy, University of Naples Federico II, Via Domenico Montesano 49, 80131 Naples, Italy; 4 Department of Pharmacy, University of Salerno, via Giovanni Paolo II 132, 84084 Fisciano (Salerno), Italy; piacente@unisa.it (S.P.); pmontoro@unisa.it (P.M.); 5 Department of Agricultural Sciences, University of Naples Federico II, Via Università 100, 80055 Portici (Naples), Italy; riccardo.aversano@unina.it (R.A.); vincenzo.damelia2@unina.it (V.D.)

**Keywords:** pigmented potatoes, potato tuber extract, leukemia cells, cell cycle modulation, bioactive molecules, potato genotyping

## Abstract

Plants produce a vast array of biomolecules with beneficial effects for human health. In this study, polyphenol and anthocyanin-rich extracts (PAE) from pigmented tubers of *Solanum tuberosum* L. varieties “Blue Star”, “Magenta Love”, and “Double Fun” in comparison with the more extensively studied “Vitelotte” were evaluated and compared for antiproliferative effects in human leukemia cells, and their phytochemical and genetic profiles were determined. In U937 cells, upon treatment with PAE, it was possible to reveal the expression of specific apoptotic players, such as caspase 8, 9, 3, and poly (ADP-ribose) polymerase (PARP), as well as the induction of monocyte and granulocyte differentiation. A liquid chromatography/electrospray ionization tandem mass spectrometry (LC-ESI-MS/MS) investigation revealed the presence of polyphenolic compounds in all the varieties of potatoes analyzed, among which caffeoyl and feruloyl quinic acid derivatives were the most abundant, as well as several acylated anthocyanins. Each pigmented variety was genotyped by DNA-based molecular markers, and flavonoid-related transcription factors were profiled in tubers in order to better characterize these outstanding resources and contribute to their exploitation in breeding. Interesting biological activities were observed for “Blue Star” and “Vitelotte” varieties with respect to the minor or no effect of the “Double Fun” variety.

## 1. Introduction

The cultivated potato (*Solanum tuberosum* L.) represents an irreplaceable staple food due to the high nutritional value and versatile use of its tubers. Consumers know that potato tubers provide a great amount of carbohydrates, but most of them ignore the fact that many varieties of potatoes are also rich in dietary fiber, potassium, ascorbic acid, protein, and several phytochemicals, above all phenolic acids. Among these latter phytochemicals, chlorogenic acids (3-, 4-, and 5-*O*-caffeoylquinic acids) are the most common, together with caffeic acid, ferulic and *p*-coumaric acids, and flavonoids such as quercetin, kaempferol, rutin, and catechin [1,2]. These compounds are powerful antioxidants with several beneficial effects on human health. Various studies demonstrated that the genetic makeup of a potato variety has more effect on the phenolic content than the environment [3,4]. Particularly attractive are purple-fleshed potatoes, in that they contain higher amount of phenolics as compared to white-fleshed ones [5]. The color of pigmented varieties is due to the presence of additional specific phenolic molecules, called anthocyanins [6,7,8]. In nature, anthocyanins provide pigmentation to different plant organs (flowers, fruits, leaves, and seeds), and are particularly abundant in black or blue colored fruits and vegetables, as well as in their derivatives (e.g., red wines). These molecules belong to the class of flavonoids, which are under study as functional food ingredients for their potential beneficial effects on human health [5,9]. Epidemiologic studies suggest that the consumption of anthocyanins contributes to maintaining human health and lowering the risk of diabetes, arthritis, and cancer; these effects are due, at least in part, to their antioxidant and anti-inflammatory activities [10]. Besides, given the many interesting biological activities, pigmented foods have a huge potential for the extraction of natural colorants and antioxidants, which are useful for developing new nutraceuticals [11].

Many in vitro studies have revealed the potential beneficial health effects of pigmented tuber extracts [3,6,7]. Pigmented potatoes display two to three times higher antioxidant potential than white-fleshed potato, and a synergic effect of the mixture of anthocyanins and other antioxidants contained in potato tubers has been demonstrated [12,13]. Additionally, highly pigmented potatoes tend to have reduced glucose responses and glycemic index values compared to white or yellow varieties [14]. The composition and biological properties of pigmented potato tubers are related to the type of anthocyanins, other phenolics, and their synergistic effects. The activation and deactivation of a specific biochemical branch of the phenylpropanoid pathway is highly regulated at the transcriptional level by several regulatory genes. Among them, members of the R2R3-MYB family are particularly important [15,16]. *StAN1* and *StAN2* (also known as *StMYBA1*), for example, impact the production of hydroxycinnamic acid derivatives and anthocyanins, while *StMYB12A* and *StMYB12B* are considered to be potentially associated with the flavonol branch [17,18]. With the recent development of genomic tools, it has become clear that the knowledge of these pathway regulators can pave the way to effective breeding strategies devoted to activating specific branches of the polyphenol pathway (e.g., those leading to anthocyanins, flavonols, and chlorogenic and ferulic acids) [9].

Only a few studies have focused on the anticancer properties of purple-fleshed potato varieties [6,7]. In light of this, the aim of our study was to evaluate and compare the antiproliferative effects of polyphenol and anthocyanin-rich extracts (PAE) obtained from tubers of four pigmented potato varieties (namely “Blue Star”, “Double Fun”, “Magenta Love”, and “Vitelotte”) in the hematological cancer cell lines U937, NB4, and HL60. The cellular and molecular mechanisms of PAE-induced anticancer activity were also investigated in U937 cells. A phytochemical analysis of the four extracts by LC-ESI-Orbitrap-MS analysis was also performed. In addition, since the content of phytochemicals in potato has a strong genetic basis, molecular genotyping was carried out. The antiproliferative activities of “Blue Star”, “Double Fun” and “Magenta Love” are described here for the first time, as no data on the biological properties of these potato varieties exist in literature.

## 2. Results

### 2.1. Polyphenol/Anthocyanin-Rich Extracts from Pigmented Tubers of Different Potato Varieties Induce Antiproliferative and Apoptotic Effects in Hematological Cancer Cell Lines

Cell viability was preliminarily tested by trypan blue assay in all cell lines at three different concentrations of PAE (1.25, 2.5, and 5 mg/mL) and up to three days after treatment by observation each 24 h. As a result, the incubation time of 48 h and the concentration of 2.5 mg/mL were selected for analysis of cell morphology. Indeed, the effects of proliferative blocking in the cell cultures were clearly observed in these conditions by optic microscopy and by occurrence of a preG1 peak, which were evaluated by cell cycle analysis (fluorescence-activated cell sorting, FACS). The analysis of cell morphology showed that all PAE have a significant antiproliferative activity on the cellular lines U937, NB4, and HL60. A greater effect was observed after treatment with “Vitelotte” and “Blue Star” PAE at the concentration of 2.5 mg/mL, as displayed by the reduced number of cells with normal morphology and the appearance of apoptotic events, such as nuclear fragmentation (Figure S1). All cancer cell lines responded to the treatment with PAE, displaying different sensitivities. The highest antiproliferative efficacy was detected on human acute myeloid leukemia (AML) U937 cell line. Consequently, the following assays were carried out on U937 cells at the intermediate concentration of 2.5 mg/mL PAE from the four pigmented varieties.

**Figure 1:**
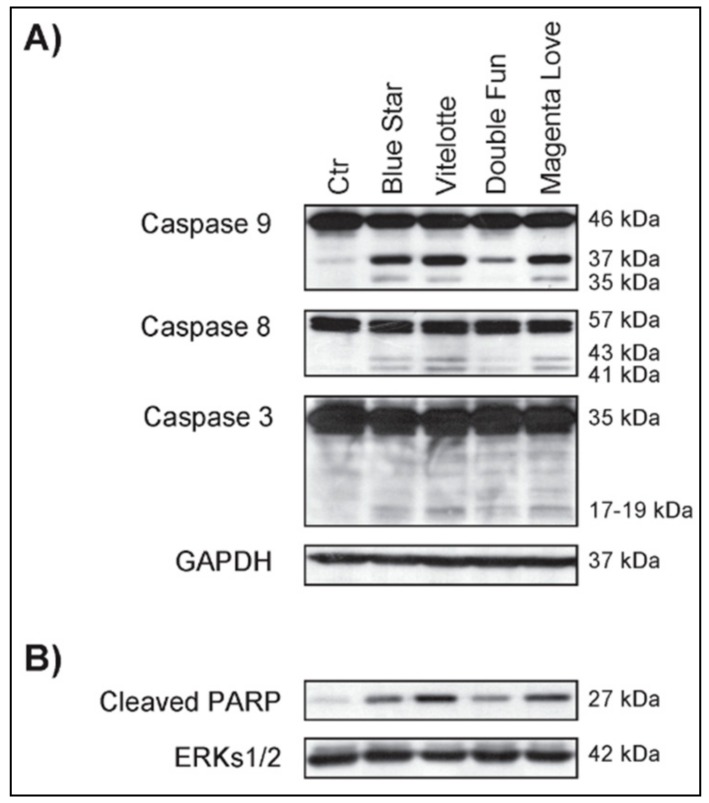
Polyphenol and anthocyanin-rich extracts (PAE) from *S. tuberosum* varieties restored the apoptotic program in U937 cancer cells, as compared to untreated U937 control cells (Ctr). (**A**) Western blot analysis of the indicated proteins in U937 cells after PAE treatment from “Magenta Love”, “Blue Star”, “Double Fun”, and “Vitelotte” varieties at 2.5 mg/mL for 24 h. Glyceraldehyde 3-phosphate dehydrogenase (GAPDH) detection was used as loading control. (**B**) Western blot analysis of the indicated protein in U937 cells after PAE treatment at 2.5 mg/mL for 24 h. Extracellular signal-regulated protein kinases 1 and 2 (ERKs 1/2) were used as loading control.

To compare the potential anticancer effects shown, and related to the restoration of apoptotic program, caspases and poly(ADP-ribose) polymerase (PARP) cleavage and activation were evaluated after treatment with PAE. U937 cells responded with apoptosis to treatment with all PAE after 24 h of treatment (Figure 1).

**Figure 2:**
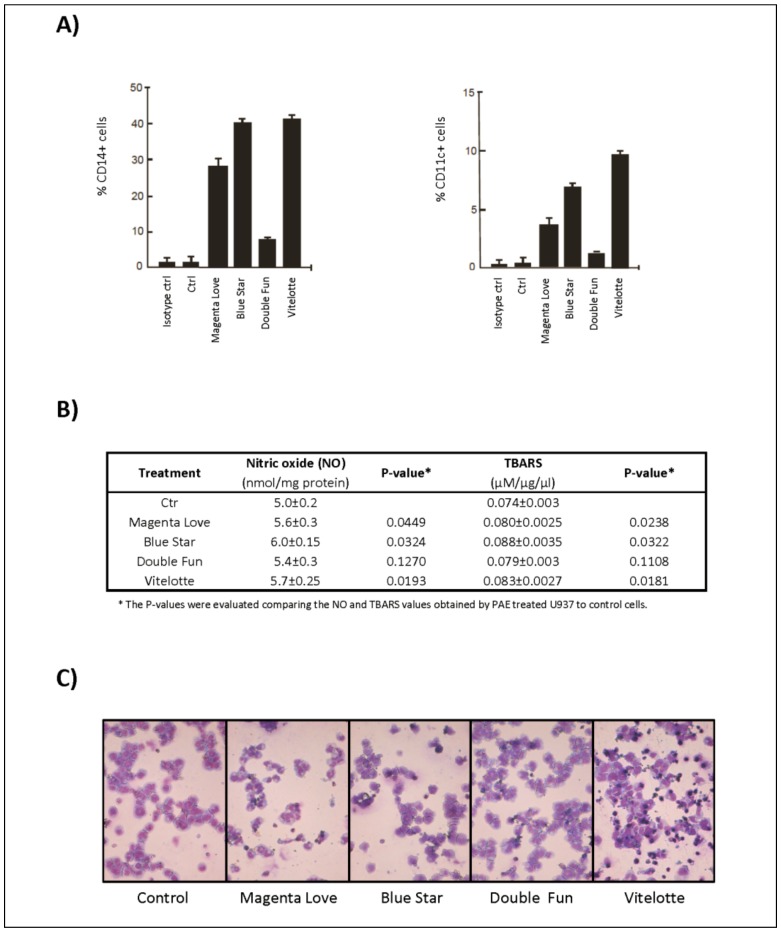
PAE from *S. tuberosum* varieties induced hematological cancer cell differentiation. (**A**) Fluorescence-activated cell sorting (FACS) analysis of CD14 (left) and CD11c (right) expression in U937 cells upon PAE treatment from “Magenta Love”, “Blue Star”, “Double Fun”, and “Vitelotte” at 2.5 mg/mL for 24 h, as compared to untreated control cells (Ctrl). Error bars represent the standard deviation from two independent experiments carried out in duplicate. Isotype controls (ctrl) were used as negative control. (**B**) Oxidative stress markers evaluated in the media (NO) and homogenates (thiobarbituric acid reactive substances, TBARS) of U937 cells treated with 2.5 mg/mL of PAE for 24 h, as compared to untreated control cells (Ctr). (**C**) Morphological analysis of granulocytic differentiation in HL60 hematological cancer cell line after treatment with 1.25 mg/mL PAE for 6 days.

Western blot analysis revealed the proteolytic cleavage for caspases 9, 8, and 3 with the production of intermediate fragments of p35/p37 kDa, p41/p43 kDa, and p18 kDa, respectively (Figure 1A). “Blue Star”, “Vitelotte”, and “Magenta Love” showed a higher activity than “Double Fun”. To support these findings, the involvement of poly (ADP-ribose) polymerase (PARP), a caspase substrate considered to be a hallmark of apoptosis, was analyzed. Results showed that the level of the p27 kDa cleaved fragment of PARP was significatively increased after PAE treatments (Figure 1B). The antiproliferative effects of the different PAE were compared also to verify the activation of differentiation pathways. With this aim, in U937 cells we studied the action of PAE on the expression of CD11c and CD14, markers of granulocyte and monocyte differentiation, respectively (Figure 2A). All PAE induced maturation-related pathways. PAE of “Magenta Love”, “Vitelotte”, and “Blue Star” were able to induce consistent monocyte differentiation and weak granulocyte maturation with respect to the minor effects from the “Double Fun” variety.

We also evaluated the free nitric oxide (NO) concentration and thiobarbituric acid reactive substances (TBARS) values in the media and cytosol, respectively, of U937 cells treated for 24 h with 2.5 mg/mL PAE of the four selected varieties (Figure 2B). We observed a significant increase of free NO and TBARS values in “Blue Star”, “Vitelotte”, and “Magenta Love” treated cells compared to control cells. Consistent with what we observed in the differentiation assay, a minor effect was observed in “Double Fun” treated cells. The higher values were related to the great expression of CD11c and CD14 markers of granulocyte and monocyte differentiation, respectively. In support of the differentiative activity induced by PAE, HL60 cells also responded to the treatment with a remarkable and morphologically evident granulocytic differentiation upon treatment with 1.25 mg/mL PAE for six days (Figure 2C). “Magenta Love” was the variety showing the greatest percentage of cells with a clear and evident granulocytic morphology.

### 2.2. MS Analyses of Polyphenol/Anthocyanin-Rich Extracts from Pigmented Tubers

**Figure 3:**
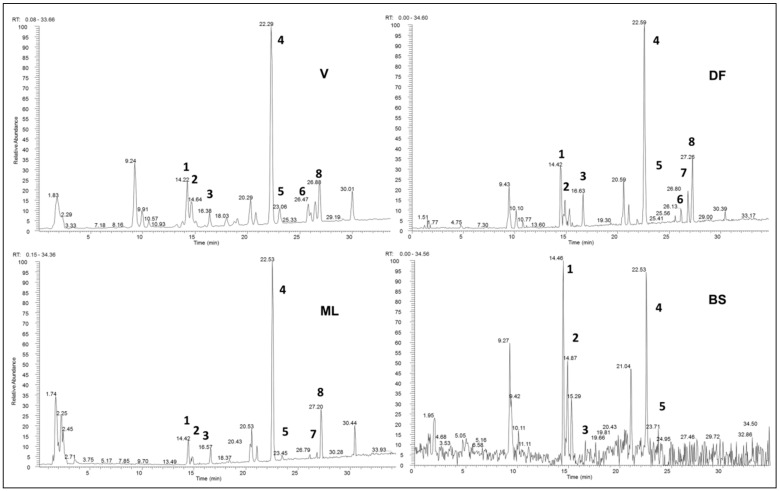
LC-ESI-Orbitrap-MS profiles of polyphenol and anthocyanin-rich extracts from pigmented tubers in negative ion mode. Note: V = “Vitelotte”; DF = “Double Fun”; ML = “Magenta Love”; BS = “Blue Star”.

PAE obtained from the different potato varieties were analyzed by LC-ESI-Orbitrap-MS and LC-ESI-Orbitrap-MS/MS in negative ion mode. The negative LC-MS profile highlighted the presence of a large group of compounds corresponding to the deprotonated molecular ions of different phenolic compounds. PAE of the different varieties showed different chromatograms when analyzed by LC-ESI-Orbitrap-MS (Figure 3).

High-resolution (HR) MS^n^ detection (positive and negative ionization modes) was used to obtain information on the structural features and the conjugated forms of phenolic compounds. Identification of compounds was based on retention times, accurate mass measurements, MS/MS data, exploration of specific metabolites from a public repository of mass spectral data (MassBank), and comparison with data reported in the literature [6,19,20]. The molecular formulas of the compounds are summarized in Table 1. LC-ESI-Orbitrap-MS/MS experiments were carried out in data-dependent scan mode, in order to select and submit the pseudomolecular ions to fragmentation experiments using the optimized parameters for the trap.

Compounds **1**, **2**, **3**, **5**, **6**, and **7** were identified by using KNApSAck database. In particular, compounds **1** and **2** are caffeoylquinic acids, which were reported in *S. tuberosum* along with feruloylquinic acid derivatives in a recent paper by Narvaez-Cuenca et al. (2013) [20]. Compound **3**, putatively identified as tuberonic acid glucoside, based on the pseudomolecular ion *m/z* value of 387.1025, mass fragmentation, and comparison with the database KNApSAck, was previously detected in *S. tuberosum* [21]. Compound **4** was putatively identified as feruloylquinic acid, based on its accurate mass determination, its mass fragmentation, and comparison with literature data [20]. This compound was found to be the main component in “Vitelotte”, “Double Fun”, and “Magenta Love” samples. In “Blue Star”, the amount of this compound was comparable to that of 3-*O*-caffeoyl-quinic acid (Table 1). Compounds **5**, **6**, and **7** are glycosylated derivatives of quercetin, which was previously detected in potato [19]. Compound **8** was identified as a sulphate derivative of quercetin; in this case, the putative identification was performed with the help of MassBank, a public repository for sharing mass spectral data [22]. It is worth noting that “Blue Star” was the only variety where quercetin derivatives were not detected but 3-*O*-caffeoylquinic acid was present in higher amounts with respect to the other potato varieties.

To detect anthocyanins, a selective multiple reaction monitoring (MRM) experiment was performed by using an LC-ESI-Q-Trap-MS/MS equipped with a quadrupole linear trap analyzer. The transition observed during ESI/MS/MS experiments on cyanidin-3-*O*-glucoside led us to predict similar fragmentation patterns for the other compounds belonging to the same class. The precursor–product transitions involved in the MRM method and the compounds detected in the different PAE samples are reported in Table 2.

In order to obtain accurate data concerning the amounts of phenolic compounds in the different *S. tuberosum* varieties, quantitative analyses were carried out for both phenolic compounds and anthocyanins (Table 1 and Table 2).

### 2.3. Molecular Genotyping of Pigmented Potato Varieties

**Figure 4:**
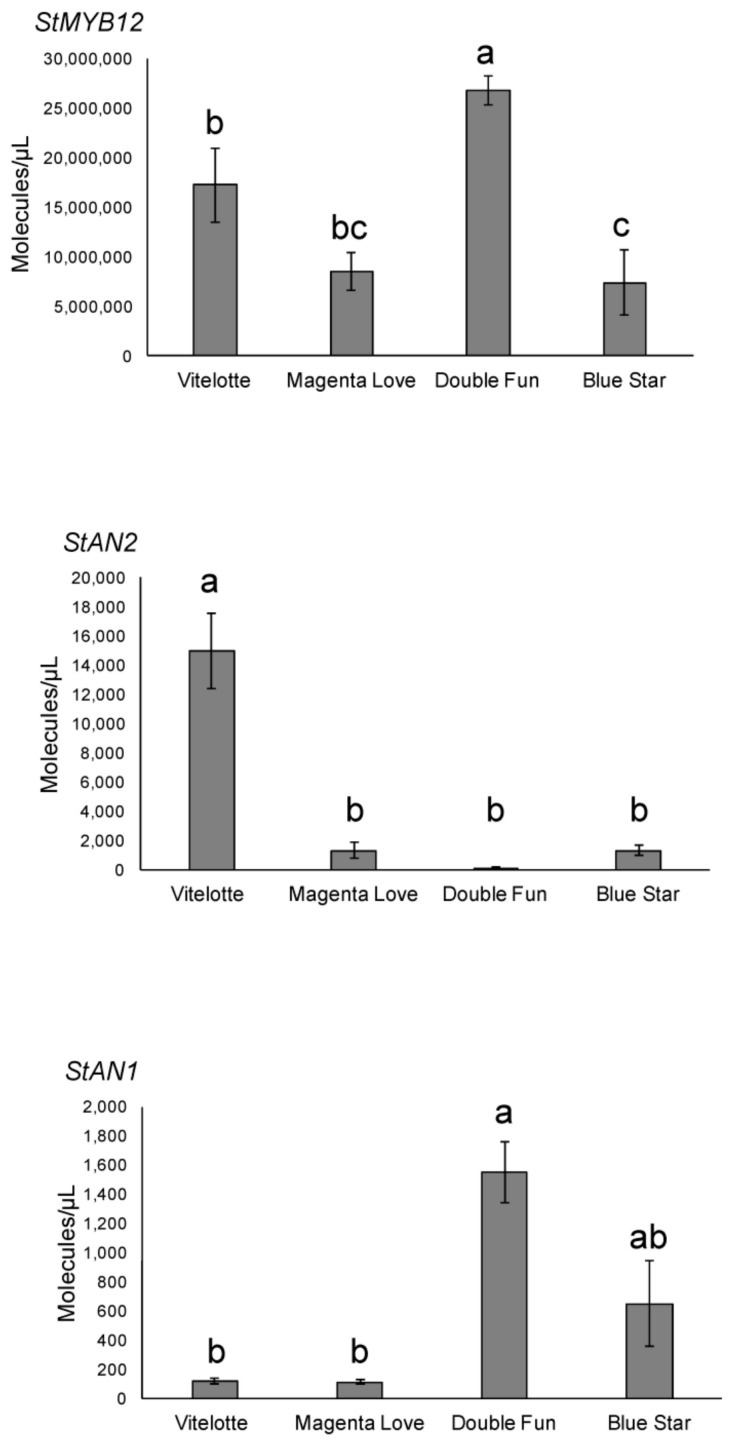
Comparative genetic profiling of pigmented potato cultivars. Gene expression analysis in tubers of potato genotypes as monitored by absolute Real-Time Quantitative Reverse Transcription PCR (qRT-PCR). Each value represents the mean of three determinations (± SD). Means denoted by the same letter did not differ significantly at *p* ≤ 0.05 according to Tukey’s test.

Simple sequence repeat (SSR) markers and transcript levels of main phenolic gene regulators were studied. A total of 11 SSR loci were successfully amplified in the four pigmented varieties, generating a total of 59 reliable alleles (Table S1), corresponding to an average of 5.36 alleles/locus. Among polymorphic alleles, we identified eight genotype-specific (private) alleles in “Blue Star”, six in “Magenta Love”, four in “Vitelotte”, and three in “Double Fun” (Table S1). Comparisons with the SSR database owned by our research group [23] indicated that the four varieties studied here tend to cluster apart from most of the varieties included in the database, highlighting their genetic uniqueness (Figure S2). For transcript analysis, we studied two homologous potato candidates of the tomato (*Solanum lycopersicum*) and *Arabidopsis thaliana* transcription factor *MYBs*, named *MYB12* and *MYB111*, which are flavonol-specific regulators of flavonoid biosynthesis [24]. We also carried out an absolute quantification of the known flavonoid regulatory genes in potato tubers *StAN1* and *StAN2* (Figure 4). Compared to the other transcription factors, *StMYB12* showed a highest abundance of transcripts in all genotypes analyzed (in the range of millions of molecules/μL). By contrast, no amplification was obtained for *StMYB111* (data not shown). The transcript abundance of *StAN2* and *StAN1* was quantified in the order of thousands (Figure 4). “Vitelotte” was the variety with the highest expression of *StAN2* (about 15,000 molecules/μL), whereas a transcript abundance below 2000 molecules/μL was monitored in the other three varieties. *StAN1* showed a general transcript abundance lower than the other two transcription factors. In contrast to *StAN2*, *StAN1* was less expressed in “Vitelotte” and highly expressed in “Double Fun” and “Blue Star”.

## 3. Discussion

### 3.1. Comparative Bioactive Effects and Characterization of PAE from Pigmented Potato Tubers

An ever-growing interest in functional foods has stimulated the study of pigmented potatoes for their potential effects on human health related to their richness in phenolic compounds, particularly anthocyanins. To increase knowledge on their healthy properties and to promote the development of cultivars with nutraceutical properties, one of the main purposes of this study was to compare the biological activity of polyphenol/anthocyanin-rich extracts (PAE) derived from tubers of the pigmented varieties “Blue Star”, “Double Fun”, and “Magenta Love” with respect to the more extensively studied variety “Vitelotte”. In previous works, we characterized the biological activities of anthocyanin extract (AE) derived from “Vitelotte” in solid and hematological cancer models [6,7], providing clear evidence that AE induced the differentiation and apoptotic pathways, and described the main molecular mechanisms involved. Here, we characterized the effects of PAE as being more representative of real metabolite composition in pigmented tubers than the previously studied AE. We compared and evaluated the potential anticancer activity of PAE from four varieties in myeloid leukemia cell lines and found differences in antiproliferative efficacy. In addition, the cellular morphology analysis displayed dose dependence from PAE and different sensitivities of the used cancer cell lines. We used extract concentrations of the order of mg/mL, as previously tested in our works [6,7], because lower concentrations did not induce significant biological effects on cancer cell lines as regards proliferative arrest, induction of apoptosis, and differentiation. The most important outcomes were obtained using “Vitelotte” and “Blue Star” varieties (Figure S1). This study also showed that the anticancer potential of the different PAE was related to the induction of apoptosis, as already demonstrated in our works on AE from “Vitelotte”, where caspase activation [6] and TNF-related apoptosis-inducing ligand (TRAIL) induction were observed [7]. After treatment with PAE, cell line U937 responded to the activation of apoptosis pathways by triggering the activation of caspases 9, 8, and 3. Our findings also provided evidence that the levels of cleaved caspases 9, 8, and 3 were upregulated. Caspases 9 and 8 are initiators of programmed cell death, while caspase 3 is an effector of apoptosis. Both the extrinsic and intrinsic pathways were activated, but the increased activation of caspase 9 suggested that the mitochondrial apoptotic pathway was mainly restored. In addition, the generation of cleaved-PARP, considered characteristic of apoptosis as the ultimate target of the caspase cascade, was significantly increased; this also confirms the proapoptotic properties of PAE. To further support these outcomes, we showed that several secondary metabolites of plant food extracts stimulated tumor cell differentiation, restoring the apoptotic program [25].

To verify whether the antiproliferative activity of PAE was accompanied by the activation of maturation-related pathways, the differentiation process to monocyte and granulocyte pathways was evaluated in U937 cells. Results indicated that PAE was able to induce monocyte differentiation and granulocyte maturation pathways, suggesting that the induction of differentiation might contribute to PAE-mediated anticancer action (Figure 2A). Important effects were observed with PAE from “Magenta Love”, “Blue Star”, and “Vitelotte”. The differentiative activity shown after treatment with PAE was also confirmed by morphological analysis of HL60 cells—used as a model of potential granulocytic maturation—which responded with an evident differentiation (Figure 2C).

In the same experimental conditions, we successively demonstrated that treatment of U937 cells with “Magenta Love”, “Blue Star”, and “Vitelotte” PAE significantly induced an increase of free nitric oxide (NO) release, while this effect was not observed for “Double Fun” PAE-treated cells. NO is an intercellular messenger that is generated by the NO synthase (NOS) enzyme and regulates many functions, such as cell cycle arrest, differentiation, and apoptosis. However, when both superoxide anions and NO are produced, they will combine spontaneously to form peroxynitrite (ONOO^−^). In vivo, peroxynitrite could induce pathogenic mechanisms, such as chronic heart failure, diabetes, circulatory shock, chronic inflammatory diseases, cancer, and neurodegenerative disorders. It is known that free NO potently inhibits the growth of myeloblastic leukemia cells [26] and induces monocytic differentiation [27]. Different studies demonstrated that the product of lipid peroxidation acts as an intracellular signal able to modulate gene expression, cell proliferation, differentiation, and apoptosis. Furthermore, the capacity of some metabolites (i.e., anthocyanins) was reported in the literature to increase NO bioavailability [28]. Some anthocyanins, such as petunidin, malvidin, and pelargonidin, possess a high capacity to scavenge superoxide anion radicals [29]. Therefore, they can increase the bioavailability of nitric oxide, which can freely cross the membrane. Guo et al. (2012) [30] demonstrated that anthocyanins functionally regulate hepatic lipid homeostasis in vitro by increased fatty acid metabolism, with consequent increase of secondary lipid oxidation products, such as malondialdheide and 4-hydroxynonenal. These reactive aldehydes were able to inhibit proliferation and to induce differentiation of murine erythroleukemia (MEL) cells [31]. Here, we showed that “Magenta Love”, “Blue Star”, and “Vitelotte” PAE treatment of U937 cells significantly increased the aldehyde concentration, which was evaluated by Thiobarbituric Acid Reactive Substances (TBARS) assay compared to control cells. The different response of “Double Fun” PAE on both NO and TBARS values may be due to the different compositions in secondary metabolites, such as anthocyanins and other phenolic compounds present in the extract.

Following the determination of the biological activity of PAE from our varieties, the next step was the phytochemical characterization of the extracts by LC-ESI-Orbitrap-MS analysis. In fact, because antiproliferative effects of PAE are the result of the synergistic action of phytochemicals contained in the pigmented tubers, it was essential to ascertain the main compounds of each variety. “Blue Star”, “Double Fun”, “Magenta Love”, and “Vitelotte” displayed the same phenolic acid pattern, with chlorogenic acids **1**, **2**, and feruloylquinic acid **4** being the main phenolic acids detected (Table 1). This is consistent with previous reports on the tuber phenolic profiles of potato varieties [1,3]. Feruloylquinic acid **4** was the main compound in “Vitelotte”, “Double Fun”, and “Magenta Love” samples. In “Blue Star”, the amount of this compound was comparable to that of 3-*O*-caffeoylquinic acid **1** (3-CQA or neochlorogenic acid). Also, in agreement with previous reports, 3-CQA **1** was the primary chlorogenic acid form in all the varieties, followed by 5-*O*-caffeoylquinic acid **2** (5-CQA or chlorogenic acid). Quercetin derivatives **5**–**8** were found in all the samples except for “Blue Star”. We did not detect catechins in any of the four samples analyzed, even if this class of compounds has been previously detected in different potato varieties [1]. Differences may be due to several factors, including methodological, agronomic, and genetic variation. The identified anthocyanins were glycosides of pelargonidin, cyanidin, malvidin, delphinidin, peonidin, and petunidin acylated with *p*-coumaric and ferulic acids, confirming that generally, colored-fleshed potatoes contain a variety of acylated anthocyanins and low levels of simple glycosides [1]. Petunidin derivatives predominated in purple potatoes (“Double Fun” and “Blue Star”), whereas pelargonidin derivatives predominated in red potatoes (“Magenta Love”), as already observed before [12]. Malvidin derivatives were not detected in the red potatoes. “Vitelotte” contained the highest variety of anthocyanins, with a prevalence of petunidin 3-*p*-coumaroylrutinoside-5-glc and malvidin 3-*O*-(6-*O*-(4-*O*-feruloyl-α-rha)-β-glc)-5-β-glc, in agreement with previously reported data [6,7]. Overall, our results show that even varieties with very similar colors can vary in anthocyanin profile.

The antiproliferative activity against human leukemia cells and several other tumor cell lines of phenolic compounds identified in our total tuber extracts has been extensively described previously, as reviewed by Miceli et al. [25]. However, it has been widely demonstrated that biological effects of polyphenol-rich extracts are the result of the synergistic action of phenolic compounds, and that the observed benefits of a polyphenol and anthocyanin-rich diet are due to the complex mixture of metabolites [32]. Data obtained in this study revealed a significant genotype influence on cytotoxic activity of PAE extracts, and this effect could be ascribed to the peculiar phenolic pattern of each potato variety.

### 3.2. Comparative Genetic Profiling of Pigmented Potato Varieties

According to Niedzwiecki et al. (2016) [32], the use of polyphenol combinations is a more effective strategy against cancer than the use of a single type of phenolic molecule. This raises the question: is it possible to develop a potato variety possessing a polyphenolic “bouquet” that exerts the best inhibitory effects on human leukemia cells? Genomics-based breeding strategies involving a detailed knowledge of genomes and genes may help to reach this goal. With this in mind, we performed both SSR marker analysis and targeted regulatory gene expression studies to determine useful molecular tools and information for the aforementioned strategy [33]. SSR markers were able to genetically differentiate our samples, traditionally distinguished by morphologic characters known to be strongly influenced by growth conditions. They also allowed the identification of private alleles (i.e., genotype-specific), which will be particularly interesting for reliable variety identification and marker-assisted selection in future breeding programs, where our varieties will be used as parental lines to produce genetic variability. The findings of high genetic diversity and genotype-specific SSR markers support the use of this genetic material in potato breeding using variety-specific information. Complementarily to SSR analysis, the expression level of regulatory *MYBs* was studied to verify if the differential expression of these genes was an important factor in determining varietal differences in bioactive molecule content. Several studies (reviewed in Liu et al., 2018 [34]) reported that in potato, *StAN1* codifies for a *MYB*-controlling anthocyanin biosynthesis in the tuber, and that *StAN1* alleles contribute with different levels of efficiency to pigment accumulation [24]. Surprisingly, “Vitelotte” produced a number of anthocyanin molecules at levels comparable to “Blue Star” and “Double Fun”, but the level of *StAN1* expression was low. We suppose that in “Vitelotte”, the function of *StAN1* is very likely substituted by *StAN2*, which we found highly expressed in this variety. *StAN2* is a *StAN1* paralog, whose function has only recently been partly clarified. *StAN2* is relatively able to activate anthocyanins but it is more efficient in activating hydroxycinnamic acid (HA) biosynthesis [18]. This probably increases the biosynthesis of organic acid precursors (i.e., ferulic and caffeic acids) that acylate anthocyanins, enhancing the antioxidant properties of these latter compounds, and consequently, their bioactivity.

Our phytochemical profiling showed that pigmented tubers have an important quantity of flavonols. In *Arabidopsis* and tomato, the branch of flavonoids leading to flavonols is controlled by two mains redundant *MYBs*, named *MYB12* and *MYB111* [35]. These genes have been scarcely studied in potato. We observed that *StMYB12* is the only one expressed in the tubers and that its expression level was lower in “Blue Star” than in the other varieties. Consistent with this outcome, “Blue Star” was the only variety where quercetin derivatives were not detected. We also found that *StMYB12* had a higher number of transcripts (millions) than *StAN1* and *StAN2*. This is consistent with the fact that *MYB12* is able to regulate metabolism at multiple levels [36]. Combining all our data, it is tempting to speculate that the contribution of flavonols (and consequently the activity of *StMYB12*) has a less beneficial impact on cancer cells studied here than the other phenolics. In fact, although no flavonols were detected in “Blue Star” tubers, its extract showed a very strong antiproliferative activity. Although further studies are needed in this regard, these genetic data may already contribute to creating tailor-made potato plants that only express the studied genes.

## 4. Materials and Methods

### 4.1. Plant Material

Commercial varieties of the common potato (*Solanum tuberosum* L.) were used in this study. They included “Blue Star” (purple skin and vascular rings), “Double Fun” (purple skin and flesh), “Magenta Love” (red skin and flesh), and “Vitelotte” (purple skin and flesh) (Figure S3). Plants were grown in pots under greenhouse conditions at the Department of Agricultural Sciences, University of Naples Federico II. Leaves of four-week-old plants were collected during growth and uniform-sized tubers were harvested at plant senescence (approximately 120 days after planting) for DNA and RNA extraction, respectively. Tubers of ~30–40 g (oval/long oval shape) were cleaned with tap water, cut into small pieces, and quickly frozen in liquid nitrogen.

### 4.2. Preparation of Crude Extracts from Pigmented Potato Tubers

The preparation of the polyphenol and anthocyanin-rich extracts (PAE) was performed according to a previous paper [37]. Uniformly sized tubers of the four potato varieties without any defects were thoroughly washed in running tap water and air dried on filter paper, cut into slices 0.5 mm in width, and then dried in a drum dryer to minimize enzymatic reactions that degrade polyphenols and anthocyanins. Next, they were ground into flakes. For preparation of pigmented extracts, 5 g of each type of flake was subjected to pigment extraction by exposure to 80% methanol (MeOH), boiled at 80 °C for 5 min, and sonicated for 20 min. The suspension was centrifuged at 5500× *g* for 10 min and extraction from the resultant precipitate was repeated under the same conditions. The MeOH in the two upper layers was combined, removed using a rotary evaporator at 35 °C, and the eluate was first dissolved in 25 mL 99.9% MeOH and then diluted to a final volume of 50 mL using distilled water. The mixture was filtered through Whatman no. 2 filter paper and stored at −4 °C until analysis.

### 4.3. Cell lines and Culture Conditions

Human leukemia cell lines U937 and HL60 were obtained from American Type Culture Collection (ATCC, Rockville, MD, USA), while NB4 cells were provided by Michel Lanotte (INSERM U-496, Centre G. Hayem Hôpital Saint-Louis, Paris, France). Cells were grown at 37 °C in 5% CO_2_ atmosphere in Roswell Park Memorial Institute (RPMI)-1640 medium (Gibco, NY, USA), then supplemented with 10% heat-inactivated fetal bovine serum (FBS), 1% l-glutamine, 1% ampicillin or streptomycin, and 0.1% gentamicin. All cell lines, initially plated at 1000 cells/mL, were treated using different dosages of PAE, corresponding to 1.25, 2.5, and 5 mg/mL, as reported for each experiment.

### 4.4. Cell Morphology Analysis

After treatment with PAE at 1.25, 2.5, and 5 mg/mL, human leukemia cell lines were spun on to glass slides using a cytospin centrifuge. Cell morphology was analyzed after May–Grünwald–Giemsa staining (Sigma-Aldrich, Saint Louis, MO, USA).

### 4.5. Nitrite Levels

Nitric oxide (NO) is rapidly converted into the stable end products nitrite and nitrate. Nitrite was measured by the Griess reaction. The human acute myeloid leukemia (AML) cells U937 were selected because they showed a higher sensitivity to PAE. The assay was carried out for 24 h at a concentration of 2.5 mg/mL PAE. Briefly, 10 μL of culture medium was mixed with an equal volume of Griess reagent (0.5% sulfanilamide, 2.5% H_3_PO_4_, and 0.05% naphthylethylene diamine in H_2_O) and incubated for 10 min at room temperature. Absorbance was assayed at 550 nm and compared with a standard curve obtained using sodium nitrite.

### 4.6. TBARS Assay

The homogenates of U937, treated for 24 h with and without 2.5 mg/mL of PAE, were incubated with 0.5 mL of 20% acetic acid solution (pH 3.5) and 0.5 mL of 0.78% aqueous solution of thiobarbituric acid. The samples were centrifuged at 4000× *g* for 5 min after heating at 95 °C for 45 min. Thiobarbituric acid reactive substances (TBARS) were quantified in the supernatant fractions by spectrophotometry at 532 nm and expressed as TBARS μM/μg of protein [38]. Each data point was the average of triplicate measurements, and each individual experiment was performed in duplicate.

### 4.7. Western Blot Analysis

Cells U937 were lysed in immunoprecipitation (IP) buffer (50 mM Tris-HCl at pH 7.0, 180 mM NaCl, 0.15% NP-40, 10% glycerol, 1.5 mM MgCl_2_, 1 mM NaMO_4_, and 0.5 mM NaF) with an added protease inhibitor cocktail (Sigma-Aldrich), 1 mM dithiothreitol (DTT), and 0.2 mM phenylmethylsulfonyl fluoride (PMSF), then kept on ice for 10 min and centrifuged at 13,000× *g* for 30 min. Protein concentration was determined in supernatant, and aliquots of 40 µg total protein extracts were separated by electrophoresis in 12% polyacrylamide gel and blotted as previously described [39,40]. Western blots were probed with antibodies against caspase 9 (#25758; 1:500; Abcam, Cambridge, UK), caspase 8 (#9746; 1:500; Cell Signaling Technology, Danvers, MA, USA), caspase 3 (#9662; 1:500; Cell Signaling Technology), and cleaved poly(ADP-ribose) polymerase (PARP) (#AF-600-NA; 1:500; R&D Systems, Minneapolis, MN, USA). Glyceraldehyde-3-phosphate dehydrogenase (GAPDH; #sc32233; 1:500; Santa Cruz Biotechnology, Dallas, TX, USA) and extracellular signal-regulated kinases 1/2 (ERKs1/2; #sc94; 1:500; Santa Cruz Biotechnology) were used to normalize for equal loading.

### 4.8. Differentiation Assay

Granulocytic and monocytic differentiation analysis was carried out as previously described [6,39]. Briefly, U937 cells, treated with and without 2.5 mg/mL of PAE, were harvested and resuspended in 10 µL phycoerythrin-conjugated CD11c (CD11c-PE) (Pharmingen, San Diego, CA, USA) or 10 µL fluorescein isothiocyanate-conjugated CD14 (CD14-FITC) (Pharmingen). Controls were resuspended with 10 µL PE- or FITC-conjugated mouse IgG1 (Pharmingen). Then, samples were incubated for 30 min at 4 °C in the dark, washed in phosphate buffered saline (PBS), and resuspended in 500 µL PBS containing 2 µL propidium iodide (PI) (Sigma-Aldrich). Samples were analyzed by FACS Calibur flow cytometer with Cell Quest technology (Becton Dickinson, San Diego, CA, USA). PI-positive cells were excluded from the analysis.

For each measurement, triplicate analyses were conducted for each sample. Differences between the means were evaluated with analysis of variance (ANOVA), using the InStat 3.0 statistic program (GraphPad Software, San Diego, CA, USA).

### 4.9. Chemicals and Sample Preparation for LC-MS Analysis

First, 1 mg of each dried potato sample was carefully weighed and dissolved in 1 mL MeOH acidified with 0.1% formic acid. The 0.1 mg/mL samples were obtained by 1:10 dilution of the prepared stock solution and were submitted to LC-MS analysis. All samples were analyzed in triplicate. Identification of compounds was confirmed by comparison with standards.; 3-*O*-caffeoylquinic acid (**1**), 5-O-caffeoylquinic acid (**2**), rutin (**6**), quercetin-3-*O*-glucoside (**7**), cyanidin-3-*O*-glucoside and quercetin-3-O-sulfate (**8**) were purchased from Extrasynthese (Lyon, France). Feruloyl quinic acid (**4**), and quercetin 3-O-(2′-glucosyl)-rutinoside (**5**) were isolated in our lab in previous studies.

### 4.10. LC-ESI-Orbitrap-MS Analysis, Qualitative and Quantitative Analyses

To investigate the main chemical markers occurring in each PAE sample, an LC-ESI-Orbitrap-MS method was developed. All experiments were performed using a Thermo Scientific liquid chromatography system, constituted of a quaternary Accela 600 pump and an Accela auto sampler connected to a linear trap–Orbitrap hybrid mass spectrometer (LTQ-Orbitrap XL, Thermo Fisher Scientific, Bremen, Germany), which was obtained by combining a linear trap quadrupole (LTQ) and an Orbitrap mass analyzer with electrospray ionization (ESI). Separation was performed on a Kinetex EVO C18 reversed phase column (150 × 2.1 mm, 5 µm, Phenomenex, Aschaffenburg, Germany). Acetonitrile, MeOH, water, and formic acid (LC-MS grade) were purchased from Merck (Darmastadt, Germany). The mobile phase consisted of solvent A (water + 1 mL/L of formic acid) and solvent B (acetonitrile/water 80/20 + 1 mL/L of formic acid). A linear gradient program at a flow rate of 0.200 mL/min was used: 0–50 min, from 0 to 40% (B); 51–56 min, 100% (B); then 0% (B) for 5 min. The mass spectrometer operated in negative ion mode. ESI source parameters were as follows: capillary voltage −12 V; tube lens voltage −121.47 V; capillary temperature 280 °C; sheath and auxiliary Gas flows (N_2_) of 30 and 5 (arbitrary units); sweep gas 0 (arbitrary units); spray voltage 5 V. MS spectra were acquired by full range acquisition covering *m/z* 200–1600 (resolution: 30,000). For the fragmentation study, a data-dependent scan was performed, selecting precursor ions corresponding to the most intense peaks in the LC-MS analysis. Data were also acquired in positive ion mode following the same chromatographic procedure. ESI source parameters were as follows: capillary voltage 49 V; tube lens voltage 120 V; capillary temperature 280 °C; sheath and auxiliary gas flows (N_2_) of 30 and 5 (arbitrary units); sweep gas 0 (arbitrary units); spray voltage 5 V. MS spectra were acquired by full range acquisition covering *m/z* 200–1600. A fragmentation study was applied also in positive mode by data-dependent scan, selecting the two most intense ions in the MS profile.

Compounds were identified according to the corresponding spectral characteristic fragmentation and retention times by comparison with data from the literature. Xcalibur software version 2.1 was used for instrument control, data acquisition, and data analysis.

Compounds **1**, **2**, **5**, and **6** were quantified by the same LC-ESI-Orbitrap-MS procedure used for qualitative analysis, using 3-*O*-caffeoylquinic acid and quercetin-3-*O*-glucoside as external standards. The other compounds were quantified on calibration curves assessed by these compounds. In particular, calibration curves were realized by means of 5 different concentration levels ranging from 0.1 mg/mL to 1 mg/mL. Quinic acid derivatives and flavonoids were quantified by means of calibration curves obtained for 3-*O*-caffeoylquinic acid and quercetin-3-*O*-glucoside, respectively.

Precision was evaluated at five concentration levels for each compound through triplicate intraday and interday assays over 3 days. Specificity was defined as the noninterference by other analytes detected in the region of interest. Linearity was evaluated by correlation values of calibration curves.

### 4.11. LC–ESI-QTrap-MS and LC–ESI-QTrap-MS/MS Analyses

UHPLC-ESI-QTrap-MS/MS (MRM) analyses were performed using an UHPLC system interfaced to an ABSciex (Foster City, CA, USA) API4000 Q-Trap instrument in ion trap mode. LC analyses were conducted using a system equipped with a Flexar UHPLC AS system (Perkin-Elmer, USA) consisting of degasser, Flexar FX-10 pump, auto sampler, and PE 200 column oven. Samples (0.005 mL) were injected into a Kinetex EVO C18 column (150 × 2.1 mm, 5 µm, Phenomenex Aschaffenburg, Germany). Acetonitrile, MeOH, water, and formic acid (LC-MS grade) were purchased from Merck (Darmastadt). Mobile phase A was H_2_O containing 1 mL/L of formic acid, while mobile phase B was acetonitrile containing 1 mL/L of formic acid. Chromatographic conditions were the same as described for the negative LC-ESI-Orbitrap-MS analysis. The flow from the chromatography was injected directly into the ESI source. The analytical parameters were optimized by infusing a standard solution of cyanidin-3-*O*-glucoside into the source at a flow rate of 0.01 mL/min. The API 6500 ES source operated in positive ion mode and the optimized parameters were: declustering potential (DP) 47.15; entrance potential (EP) 7; collision energy (CE) 26.51; collision cell exit potential (CXP) 16.3; temperature of ion source (TEM) 500 °C; nebulizing gas (GS1) 35; drying gas (GS2) 20. The column was kept at 40 °C. Data acquisition and processing were performed using Analyst software 1.6.2 (Sciex). For quantitative analyses, the Multiple Reaction Monitoring (MRM method) was applied using the precursor–product transitions assessed for qualitative analyses, with cyanidin-3-*O*-glucoside as the external standard. Calibration curves were realized by means of 5 different concentration levels in ranging from 0.1 mg/mL to 1 mg /mL. Precision was evaluated at five concentration levels for each compound through triplicate intraday assays and interday assays over 3 days. Specificity was defined as the noninterference by other analytes detected in the region of interest. Linearity was evaluated by correlation values of calibration curves.

### 4.12. Genotyping and Transcript Profiling

Genomic DNA for molecular marker profiling was isolated from 1.5 g of fully developed young leaves of two different plants for each of the four pigmented potato genotypes, as previously described by De Masi et al. (2007) [41]. DNA integrity, quality, and quantity were assessed by gel electrophoresis and spectrophotometric analysis. Genotyping was performed using 11 microsatellite (SSR) loci, previously assayed in *S. tuberosum* [42]. SSR-PCR and capillary electrophoresis were performed as reported by Bontempo et al. (2015) [7]. Two biological replicates were analyzed for each SSR locus. The alleles for SSR loci of each potato genotype were assigned by their molecular size and scored as present (1) or absent (0) by GeneScan Analysis software (Applied Biosystems, Foster City, CA, USA). SSR profiles obtained on the four pigmented potato genotypes were finally integrated with 22 further profiles from as many commercial potato varieties [23]. Genetic distances were calculated by the Dice coefficient [7] and described through a dendrogram constructed using unweighted pair group method with arithmetic mean (UPGMA) clustering using R software version 3.2.1 (18 June 2015).

Gene expression and allelic analyses were performed on flavonoid-related transcription factors (*StAN1*, *StAN2*, *StMYB12*, and *StMYB111*). Total RNA was purified from 100 mg of potato tubers of our varieties by using the Spectrum Plant Total RNA Kit and On-Column DNase I Digestion Set (Sigma-Aldrich), following manufacturer’s instructions. Here, 500 ng of total RNA were reverse transcribed to complementary DNA (cDNA) using oligo-(dT)_20_ primer and SuperScript III reverse transcriptase (Invitrogen, www.thermofisher.com) in 20 μL of final reaction according to the manufacturer’s instructions. Primer pairs used for Real-Time Quantitative Reverse Transcription PCR (qRT-PCR) of *StAN1* and *StAN2* were the same as those used by D’Amelia et al. (2018) [18]. Primers for *StMYB12* and *StMYB111* were the same as those used by Payyavula et al. (2013) [17]. For transcript profiling, an absolute qRT-PCR was performed as described in D’Amelia et al. (2014) [43]. All reactions were run in triplicate using QuantiFast SYBR Green PCR Kit (Qiagen, www.qiagen.com) in a final volume of 20 μL reactant. The qRT-PCR was carried out using the Rotor-Gene 6000 software (Corbett, www.qiagen.com) and cycle conditions were indicated by QuantiFast SYBR Green PCR Kit handbook (Qiagen). Gene expression analysis was carried out using Rotor-Gene 6000 software. The standard curves were used to calculate the copy number of molecules per μL of the corresponding target genes in each potato variety. Analysis of variance on qRT-PCR data was carried out using XLSTAT-Pro 7.5.3 software (Addinsoft, www.xlstat.com). Tukey’s test was used to compare mean values.

## 5. Conclusions

In conclusion, here we compared the antiproliferative potential of polyphenolic extracts of tubers of four pigmented potato varieties (“Blue Star”, “Double Fun”, “Magenta Love”, and “Vitelotte”) by evaluating different parameters, such as induction of apoptosis and differentiation. Our data showed many differences regarding the genetic profile, phytochemical composition, and biological activity. The most important outcomes were obtained for “Blue Star” and the more extensively studied “Vitelotte” compared to the minor or no effect of “Double Fun”. It is worth noting that “Blue Star”, whose extract showed the major antiproliferative activity, was the only variety where quercetin derivatives were not detected, but 3-*O*-caffeoylquinic acid was present in higher amounts compared to the other varieties.

The relevant biological effects of polyphenol extracts from pigmented potato varieties may be particularly advantageous from a public health perspective, considering the large food consumption of potato tubers worldwide. However, absorption, distribution, and metabolism of polyphenols and anthocyanins are still not sufficiently known aspects, and additional information is needed to define their potential useful concentrations in health protection. We are still far from determining the doses that should be consumed to obtain the maximum benefits. Furthermore, pharmacological studies should verify possible side effects from using large quantities and high concentrations of these secondary plant metabolites. We believe that further in vitro and in vivo studies on the bioaccessibility, bioavailability, and effects of potato metabolites identified here must be carried out.

## Figures and Tables

**Table 1:** High-resolution (HR) MS and MS/MS data of phenolic compounds in LC-ESI-Orbitrap-MS profiles of the PAE from the four *Solanum tuberosum* varieties.

Phenolics	Mass Parent ion (*m/z*)	MS/MS	Compound	Molecular Formula	V mg/100g DW	DF mg/100g DW	ML mg/100g DW	BS mg/100g DW
**1**	353.0149	191.0235	3-*O*-caffeoylquinic acid	C_16_H_18_O_9_	32.2 ± 2.0	32.0 ± 2.0	12.0 ± 1.0	108.0 ± 30.0
**2**	353.0867	191.0151	5-*O*-caffeoylquinic acid	C_16_H_18_O_9_	31.0 ± 1.0	21.0 ± 6.0	11.0 ± 1.0	59.0 ± 3.0
**3**	387.1025	335.2375 299.0197	tuberonic acid glc	C_18_H_28_O_9_	10.1 ± 0.06	10.7 ± 0.08	8.6 ± 0.08	6.7 ± 0.1
**4**	367.1026	191.0210 160.9260 134.8752	feruloyl quinic acid	C_17_H_20_O_9_	105.0 ± 5.9	98.0 ± 9.7	97.0 ± 8.9	107.0 ± 25.0
**5**	771.2523	720.3513 711.2075 696.3084 662.3907	quercetin-3-*O*-(2′-glucosyl)-rutinoside	C_33_H_40_O_21_	6.5 ± 0.2	ND	5.5 ± 0.1	ND
**6**	609.1450	301.1120	rutin	C_27_H_30_O_16_	13.3 ± 0.6	8.5 ± 0.6	3.8 ± 0.2	ND
**7**	463.0901	301.9224	quercetin-3-*O*-glucoside	C_21_H_20_O_12_	3.5 ± 0.2	7.8 ± 0.3	2.7 ± 0.2	ND
**8**	381.1183	301.1876 178.9705 134.8748	quercetin-3-*O*-sulphate	C_15_H_10_O_10_S	18.7 ± 0.2	29.0 ± 0.3	16.8 ± 0.2	ND

Note: DW = dry Weight; ND = not detected; V = “Vitelotte”; DF = “Double Fun”; ML = “Magenta Love”; BS = “Blue Star”.

**Table 2:** HR MS and MS/MS data for the identification of anthocyanins (An) in LC-ESI-QqQ-MS/MS profiles of the PAE from the four *S. tuberosum* varieties.

An	Mass Parent Ion (*m/z*)	MS/MS	Tentative ID	Molecular Formula	V mg/100g DW	DF mg/100g DW	ML mg/100g DW	BS mg/100g DW
**1′**	887.2609	271.0234	pelargonidin 3-(4‴-p-coumaroylrutinoside)-5-glc	C_42_H_47_O_21_	16.32 ± 0.02	14.32 ± 0.09	18.12 ± 0.04	12.08 ± 0.05
**2′**	903.2558	287.0124	cyanidin-3-(p-coumaroyl)-rutinoside-5-glc	C_42_H_47_O_22_	0.87 ± 0.03	ND	ND	ND
**3′**	917.2715	301.0051	peonidin 3-*O*-[6-*O*-(4-*O*-(E)-p-coumaroyl-O-α-rha)-β-glc]-5-*O*-β-glc	C_43_H_49_O_22_	1.23 ± 0.08	ND	ND	ND
**4′**	917.2715	301.1581	peonidin 3-caffeoylrutinoside-5-glc	C_43_H_49_O_23_	1.08 ± 0.06	ND	1.10 ± 0.07	ND
**5′**	933.2664	303.1961	delphinidin 3-*O*-[6-*O*-(4-*O*-(E)-p-coumaroyl-*O*-α-rha)-β-glc]-5-*O*-β-glc	C_42_H_47_O_23_	0.85 ± 0.03	ND	ND	ND
**6′**	919.2508	317.1110	petunidin 3-(4‴-p-coumarylrutinoside)	C_37_H_39_O_18_	ND	2.52 ± 0.03	ND	2.05 ± 0.04
**7′**	933.2664	317.0415	petunidin 3-*p*-coumaroylrutinoside-5-glc	C_43_H_49_O_23_	3.78 ± 0.03	3.28 ± 0.03	3.05 ± 0.02	3.45 ± 0.03
**8′**	977.2926	331.1865	malvidin 3-*O*-(6-*O*-(4-*O*-feruloyl-α-rha)-β-glc)-5-β-glc	C_45_H_53_O_24_	8.91 ± 0.05	6.57 ± 0.03	ND	1.02 ± 0.01
**9′**	917.2715	271.3245	pelargonidin 3-(4‴-feruloylrutinoside)-5-glc	C_43_H_49_O_22_	ND	5.21 ± 0.04	4.07 ± 0.05	0.22 ± 0.01
**10′**	949.2614	317.1456	petunidin 3-*O*-[6-*O*-(4-*O*-(E)-caffeoyl-*O*-α-rha)-β-glc]-5-*O*-β-glc	C_43_H_49_O_24_	0.32 ± 0.03	1.62 ± 0.04	ND	1.60 ± 0.03
**11′**	963.2773	317.1335	petunidin 3-*O*-[6-*O*-(4-*O*-(4-*O*-(β-D-glc)-feruloyl)-α-L-rha)-β-D-glc]- 5-*O*- [β-D-glc]	C_44_H_51_O_24_	ND	0.42 ± 0.01	ND	0.65 ± 0.04

Note: DW = dry weight; ND = not detected; An = anthocyanins; V = “Vitelotte”; DF = “Double Fun”; ML = “Magenta Love”; BS = “Blue Star”; glc = glucopyranoside; rha = rhamnopyranosyl.

## Supplementary Materials

The following are available online. Figure S1: PAE from *S. tuberosum* varieties inhibited hematological cancer cell proliferation. Figure S2: Tree diagram showing the genetic distances, calculated using SSR loci, between the four pigmented varieties and 22 commercial varieties of *S. tuberosum*. Figure S3: Tuber phenotype of the pigmented potato varieties used in this study. Table S1: Microsatellite alleles detected at SSR loci in the four pigmented potato varieties in study.
