# Supplementary material for: Comparative Phytochemical Characterization, Genetic Profile, and Antiproliferative Activity of Polyphenol-Rich Extracts from Pigmented Tubers of Different Solanum tuberosum Varieties
Source: Molecules. 2020 Jan 6;25(1):233. doi: 10.3390/molecules25010233 (PMC6983029; doi:10.3390/molecules25010233)
Supplement: Supplementary file 1 [file molecules-25-00233-s001.pdf]

SUPPLEMENTARY MATERIALS

Comparative phytochemical characterization, genetic profile and antiproliferative activity of polyphenol-rich extracts from pigmented tubers of different *Solanum tuberosum* varieties

Luigi De Masi [1,§], Paola Bontempo [2,§], Daniela Rigano [3,*], Paola Stiuso [2], Vincenzo Carafa [2], Angela Nebbioso [2], Sonia Piacente [4], Paola Montoro [4], Riccardo Aversano [5], Vincenzo D'Amelia [5], Domenico Carputo [5,*] and Lucia Altucci [2,*]

[1] National Research Council (CNR), Institute of Biosciences and Bioresources (IBBR), Via Università 133, 80055 Portici (Naples), Italy

[2] Department of Precision Medicine, University of Campania "Luigi Vanvitelli", Via L. De Crecchio 7, 80138 Naples, Italy

[3] Department of Pharmacy, University of Naples Federico II, Via Montesano 49, 80131 Naples, Italy

[4] Department of Pharmacy, University of Salerno, via Giovanni Paolo II 132, 84084 Fisciano (Salerno), Italy

[5] Department of Agricultural Sciences, University of Naples Federico II, Via Università 100, 80055 Portici (Naples), Italy

[§] The first two authors contributed equally to this research work.

* Correspondence: drigano@unina.it (D.R.); domenico.carputo@unina.it (D.C.); lucia.altucci@unicampania.it (L.A.).

SUPPLEMENTARY MATERIALS CONTENT

Figure S1. PAE from *S. tuberosum* varieties inhibited hematological cancer cell proliferation. Morphological analysis carried out in U937, NB4, and HL60 cancer cells treated for 2 days with 2.5 mg/mL of PAE from Magenta Love, Blue Star, Double Fun, and Vitelotte.

Figure S2. Tree diagram showing the genetic distances, calculated using SSR *loci*, between the four pigmented varieties and 22 commercial varieties of *S. tuberosum*.

Figure S3. Tuber phenotype of the pigmented potato varieties used in this study.

Table S1. Microsatellite alleles detected at SSR *loci* in the four pigmented potato varieties in study.

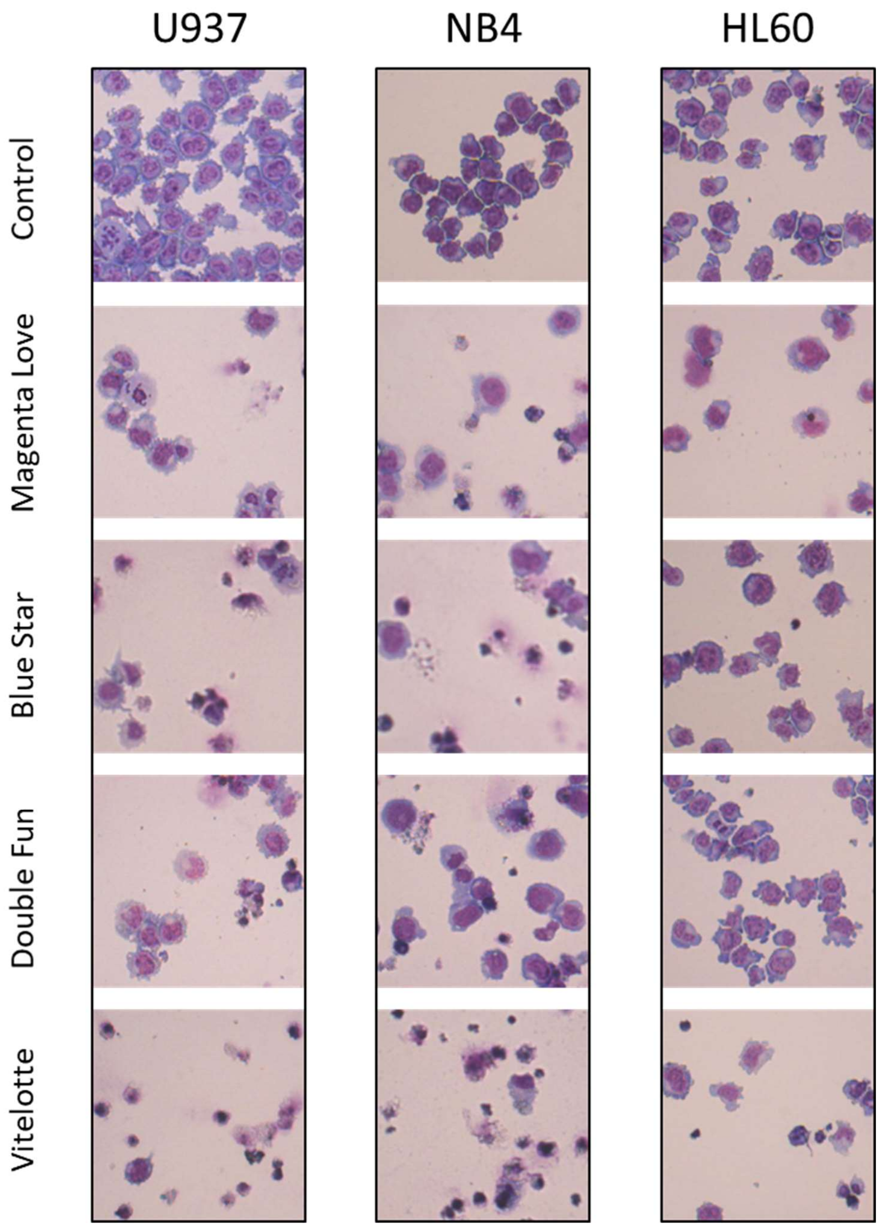

Figure S1. PAE from *S. tuberosum* varieties inhibited hematological cancer cell proliferation. Morphological analysis carried out in U937, NB4, and HL60 cancer cells treated for 2 days with 2.5 mg/mL of PAE from Magenta Love, Blue Star, Double Fun, and Vitelotte.

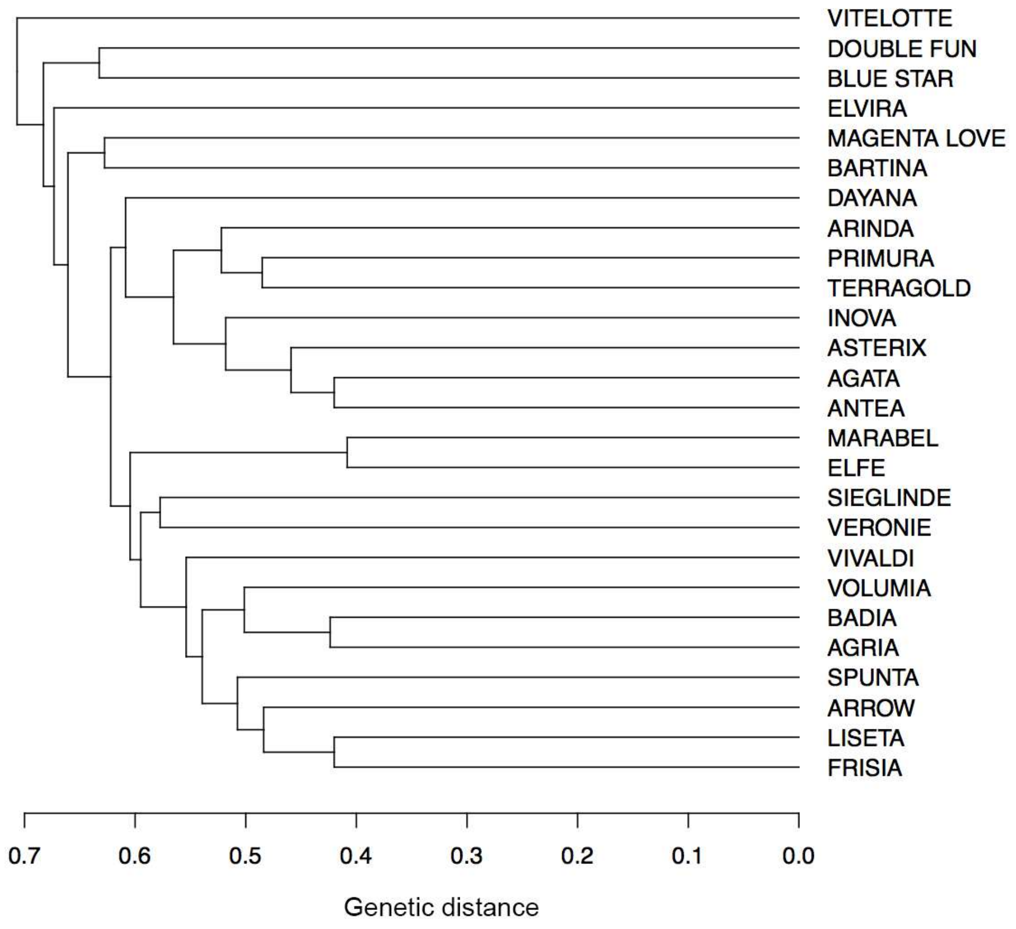

Figure S2. Tree diagram showing the genetic distances, calculated using SSR *loci*, between the four pigmented varieties and 22 commercial varieties of *S. tuberosum*. The lines linking the varieties are proportional to the estimated genetic distance.

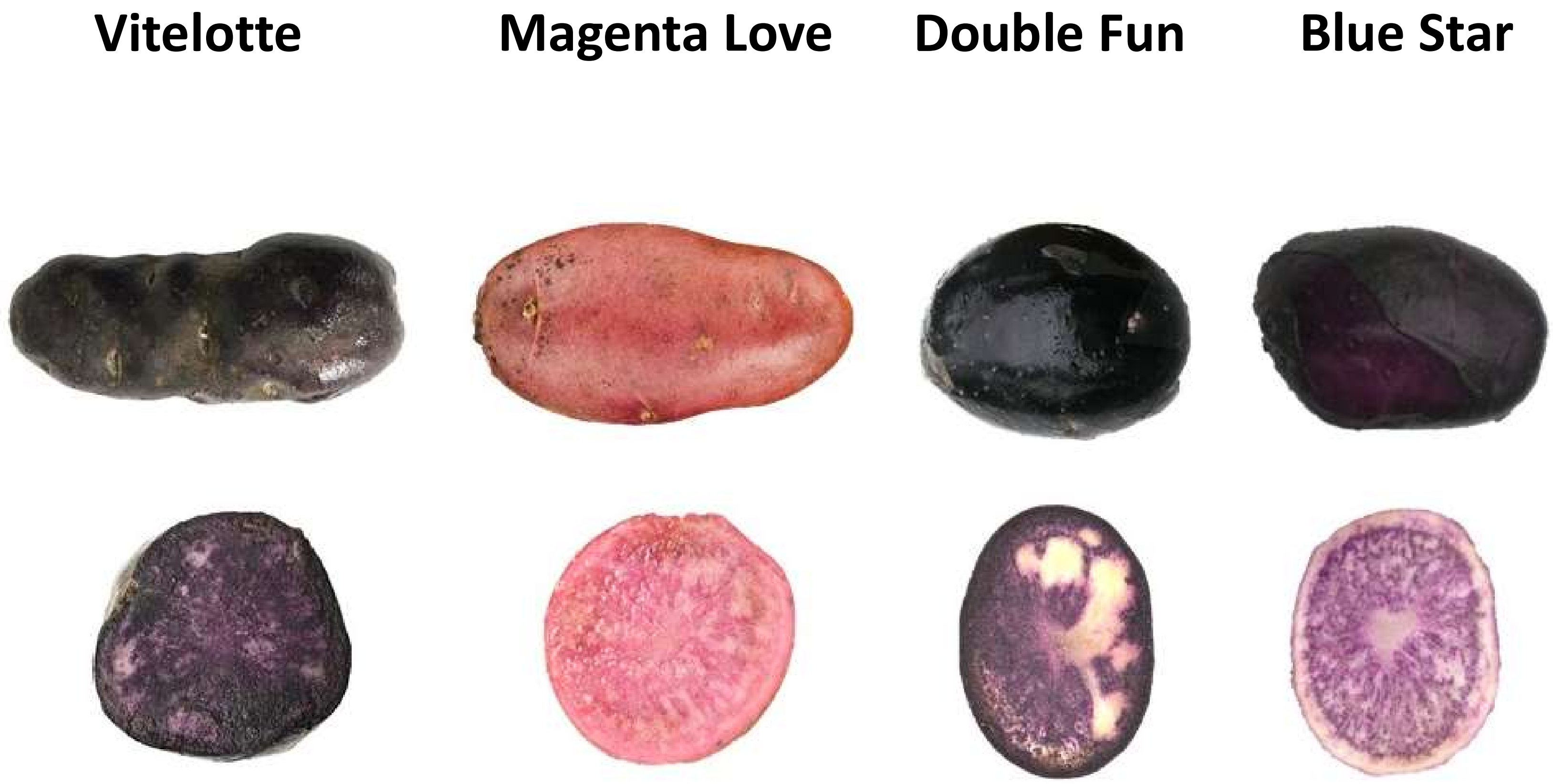

Figure S3. Tuber phenotype of the pigmented potato varieties used in this study.

Table S1

Microsatellite alleles (bp) detected at 11 SSR *loci* in the four pigmented potato varieties in study. Genotype-specific (g.s.) alleles for each variety and *locus* are reported in bold and underlined type.

Variety (reference)	STG0001	STI0004	STI0012	STI0030	STI0032	STM0031	STM1052	STM1053	STM1104	STM5114	STM5127	Total alleles (g.s.)
Blue Star (BIE02-133)	**125**, 127, 132, **143**	74, 96, 99	168, 174, **190**	83, **93**, **95**, **137**	110, 116, 119, 122	**109**, 124, 136, 187	228, **258**	169	166, 169, 173, 176	284, 286, 289	241, 244	34 (**8**)
Double Fun (RHT02-1)	**113**, **120**, 127, 132	74, 96, 99	165, 168, 174	83, 88, 104	107, 110, 119, 122	136, 187	210, 219, 228	169, 172	166, 169, 173, 176	286, 298	244, **253**	32 (**3**)
Magenta Love (VZF04-436)	**117**, 127, **135**, 139	74, **89**, 92	165, **171**, **184**	83, 88, 104	110, 116, 119, 122	124, 136, 145, 187	210, **254**	169, 172	169, 173, 182	284, 289	241, 244	32 (**6**)
Vitelotte	127, 132, 139, **145**	74, 92, 99	165, 168	**85**, 88	107, 116, 122	124, 136, 145	210, 219	172, **175**	169, 173, 176, 182	284, 289, **292**, 298	241, 244	31 (**4**)
Alleles per *locus*	10	5	6	7	5	5	5	3	5	5	3	59 (**21**)
